# Machine learning-based B cell-related diagnostic biomarker signature and molecular subtypes characteristic of ulcerative colitis

**DOI:** 10.18632/aging.205510

**Published:** 2024-02-05

**Authors:** Guo-Liang Wu, Li Li, Xiao-Yao Chen, Wei-Feng Zhang, Jun-Bo Wu, Xiaoning Yu, Hong-Jin Chen

**Affiliations:** 1 First Clinical Medical College, Nanjing University of Chinese Medicine, Nanjing, Jiangsu 210023, China; 2 Department of Anorectal Section, The First Affiliated Hospital of Shandong First Medical University, Jinan, Shandong 250014, China; 3 Department of Endocrinology, The Affiliated Hospital of Shandong University of Traditional Chinese Medicine, Jinan, Shandong 250014, China; 4 Department of Colorectal Surgery, The Affiliated Hospital of Nanjing University of Chinese Medicine, Nanjing, Jiangsu 210029, China; 5 Department of Anorectal Section, The Affiliated Hospital of Xuzhou Medical University, Xuzhou, Jiangsu 221000, China; 6 Department of Colorectal Surgery, Hengyang Central Hospital, Hengyang, Hunan 421001, China; 7 Department of Geriatrics, Hematology and Oncology Unit, Qilu Hospital of Shandong University, Jinan, China

**Keywords:** ulcerative colitis, B cell-related genes, diagnostic biomarker, immune infiltration, machine learning

## Abstract

As an inflammatory bowel disease, ulcerative colitis (UC) does not respond well to current treatments. It is of positive clinical significance to further study the pathogenesis of UC and find new therapeutic targets. B lymphocytes play an important role in the pathogenesis of UC. The effect of anti-CD20 therapy on UC also provides new evidence for the involvement of B cells in UC process additionally, suggesting the important role and potential therapeutic value of B cells in UC. In this study, we screened the most critical immune cell-related gene modules associated with UC and found that activated B cells were closely related to the gene modules. Subsequently, key activated B cell-associated gene (BRG) signatures were obtained based on WGCNA and differential expression analysis, and three overlapping BRG-associated genes were obtained by RF and LASSO algorithms as BRG-related diagnostic biomarkers for UC. Nomogram model was further performed to evaluate the diagnostic ability of BRG-related diagnostic biomarkers, subsequently followed by UC molecular subsets identification and immunoinfiltration analysis. We also further verified the expressions of the three screened BRGs *in vitro* by using an LPS-induced NCM460 cell line model. Our results provide new evidence and potential intervention targets for the role of B cells in UC from a new perspective.

## INTRODUCTION

Ulcerative colitis (UC) is a chronic inflammatory bowel disease (IBD) that causes chronic diarrhea and rectal bleeding. The incidence of UC is increasing globally and has a significant impact on life expectancy [1]. The current first-line treatment for inducing and maintaining mild to moderate UC remission is 5-aminosalicylic acid. The latest treatment for moderate to severe ulcerative colitis is oral corticosteroid-induced remission, coupled with small molecule drugs such as anti-tumor necrosis factor, α4β7 integrin and Janus kinase inhibitors to maintain remission [2]. However, in clinical trials, the highest response range of UC patients to these drug treatments was only 30% to 60%. Many patients still require hospitalization and 10–20% require colectomy [3]. Therefore, it is of positive clinical significance to further study the pathogenesis of UC in order to obtain new therapeutic targets.

B lymphocytes play a crucial role in the pathogenesis of UC. B cells are responsible for antibody synthesis, antigen delivery to T cells, and regulation of inflammatory responses by secreting cytokines such as IL-2, IL-10, IFN-γ, and TGF-β [4]. The percentage of CD23^+^ B cells increased in all UC patients [5]. There is evidence for the role of B cells in chronic inflammation, as well as abnormal B cell responses in UC patients [6]. B cells can be divided into effector cells that secrete antibodies and cytokines, and IL-10-secreting regulatory cells (Bregs) [7]. The reduction of Bregs was identified as a characteristic indicator of UC. The frequency of Bregs in peripheral blood and intestinal tissue was significantly reduced in UC patients [8]. Meanwhile, serum IL-10 levels in UC patients were negatively correlated with Mayo Clinic scores, CRP and ESR [8]. However, a clinical trial comparing the efficacy of B cell depletion therapy with placebo reported negative results, which requires further investigation of the role of B cells in UC and obtaining new relevant therapeutic targets [6].

In addition, the effect of anti-CD20 therapy on UC provides new evidence for the involvement of B cells in the UC process. As a transmembrane protein expressed in B cells, the effect of CD20-related therapy on UC has received increasing attention. Intestinal mucosal immunity depends on the balance between pro-inflammatory and anti-inflammatory stimuli of the immune system, and it has been shown that antigen presentation by B cells may be beneficial for UC [9]. B cells may have a protective effect in UC by producing the anti-inflammatory cytokine IL-10 [10, 11]. Monocytes in UC patients who were not treated with anti-CD20 rituximab (RTX) produced higher levels of IL-10 [12]. Since the clinical application of RTX, there have been reports of endogastrointestinal toxicity of UC caused by RTX [13, 14]. After stopping RTX and giving IBD-specific therapy, UC symptoms improved in most patients [15]. Anti-CD20 monoclonal antibodies lead to new onset of IBD through changes in the degree of inflammation and damage to the gastrointestinal mucosal immune environment [16]. The reduction of B cells is thought to underlie the maintenance of intestinal mucosal immune homeostasis and may underlie this adverse reaction [17]. Serum concentrations of TNF-α and IL-6 increased after RTX treatment, as did monocytes expressing TNF-α in the colon, suggesting that B cells may regulate TNF-α production in colon inflammation [18]. All these evidences suggest the important role and potential therapeutic value of B cells in UC. It has positive clinical significance for further research.

In this study, we screened the most critical immune cell-related gene modules associated with UC, and found that activated B cells were strongly correlated with gene modules. Based on WGCNA, differential expression analysis as well as RF and LASSO algorithms, key activated B cell-related gene (BRG) signatures were obtained and three BRGs were screened as UC diagnostic biomarkers. The LPS-induced NCM460 cell line model was also applied *in intro* to further verify the abnormal expressions of the three screened BRGs. Our results provide new evidence and potential intervention targets for the role of B cells in UC from a new perspective.

## MATERIALS AND METHODS

### Data download and batch effect removal

We downloaded four GEO datasets containing healthy (HC) and ulcerative colitis (UC) samples from the GEO database for the subsequent analysis. Among the four GEO datasets, GSE48634 (GPL10558, Illumina HumanHT-12 V4.0 expression beadchip) and GSE92415 (GPL13158, (HT_HG-U133_Plus_PM) Affymetrix HT HG-U133+ PM Array Plate) (90 HC and 122 UC samples) were combined as the training cohort. GSE179285 (GPL6480, Agilent-014850 Whole Human Genome Microarray 4x44K G4112F (Probe Name version)) and GSE107499 (GPL15207, (PrimeView) Affymetrix Human Gene Expression Array) (31 HC and 175 UC samples) were combined as the validation cohort. To remove the batch effect of each GEO dataset, we utilized the “SVA” package to standardize processing the transcriptome data [19, 20].

### Immune cell proportion estimation and WGCNA development

According to the 23 types immune cell signatures, we utilized the “GSVA” package to estimate the Single Sample Gene Set Enrichment Analysis (ssGSEA) score of 23 types immune cells proportion (Supplementary Table 1). Based on the “WGCNA” script, a WGNCA related model was constructed to identify the pivotal immune cell associated gene module. In first, an all-sample clustering tree was established to remove the abnormal samples and calculate the optimal soft threshold (β) to build the WGCNA. Utilizing the Pearson correlated algorithm, the potential association of each gene module and immune cells was evaluated and the most related gene module was selected for the final analysis.

### Differential expression gene (DEGs) identification and molecular function enrichment analysis

With the selection threshold set at |FC| > 1.4 and *p*.adjust < 0.05, the DEGs between HC and UC groups were identified. “clusterProfiler” package was utilized to explore the potential molecular function of GO and KEGG terms. Based on the reference of “c2.cp.kegg. v7.4.symbols”, a Gene Set Enrichment Analysis (GSEA) was carried out to predict the KEGG terms of HC and UC groups.

### Machine learning algorithm to select the feature diagnostic biomarker and nomogram establishment

Utilizing the “glmnet” package, a LASSO model was established to identify the feature variables. Then, random forest (RF) algorithm was performed to calculate the importance of each variable. The overlapping genes obtained by RF and LASSO were identified as the feature diagnostic biomarker for UC. Based on the expression profile of the diagnostic biomarker, a nomogram model was established via “glmnet” package. The nomogram score was calculated using the formula: nomogram score = CHI3L1 × 0.33 + MMP7 × 0.48 + PCK1 × −0.35. Package “pROC” was employed to evaluate the diagnostic effectiveness of biomarker for UC.

### Cell line model construction and quantitative real-time PCR (qRT-PCR)

The NCM460 cell line was cultured in RPMI-1640 medium (SH30809; HyClone, USA) supplemented with 10% fetal bovine serum (FBS; Sijiqing, Hangzhou, China) at 37°C in a humidified atmosphere containing 5% CO_2_. NCM460 cells were seeded and then treated with lipopolysaccharide (LPS) at a concentration of 1 μg/ml (Sigma, USA) for 24 hours to create an *in vitro* model of ulcerative colitis (UC) cells. After 24 hours, the LPS-induced NCM460 cells were collected for further experiments. Total cellular RNA was extracted using Trizol (Cat#9109; TaKaRa, Japan), and cDNA was synthesized using the PrimeScript RT Master Mix kit (Cat#RR047A; Takara, Japan). The mRNA levels were quantified using SYBR Green PCR Mix (Cat#RR420A; Takara, Japan). Data analysis was performed using the 2^−ΔΔCT^ method, and each experiment included three separate control sets.

### Generation of molecular subgroups pattern

According the expression profile of diagnostic biomarker, a molecular subgroup pattern of UC samples was generated using “ConsensusClusterPlus” package. Based on the optimal classification of K = 2–9, the UC samples were divided into different molecular subgroups.

### Statistical analysis

Data analysis and processing in this study were performed in R language environment. Correlation analysis between the two groups was calculated using Pearson algorithm. Statistical differences between the two groups were calculated using Wilcox rank-sum test. *p* < 0.05 was considered statistically significant. ^*^p < 0.05. ^**^p < 0.01, ^***^p < 0.001.

### Data availability statement

The datasets generated and/or analyzed during the current study are available in the repositories of GEO.

## RESULTS

### Generation of the pivotal activated B cell-related gene module for UC sample

**Figure 1:**
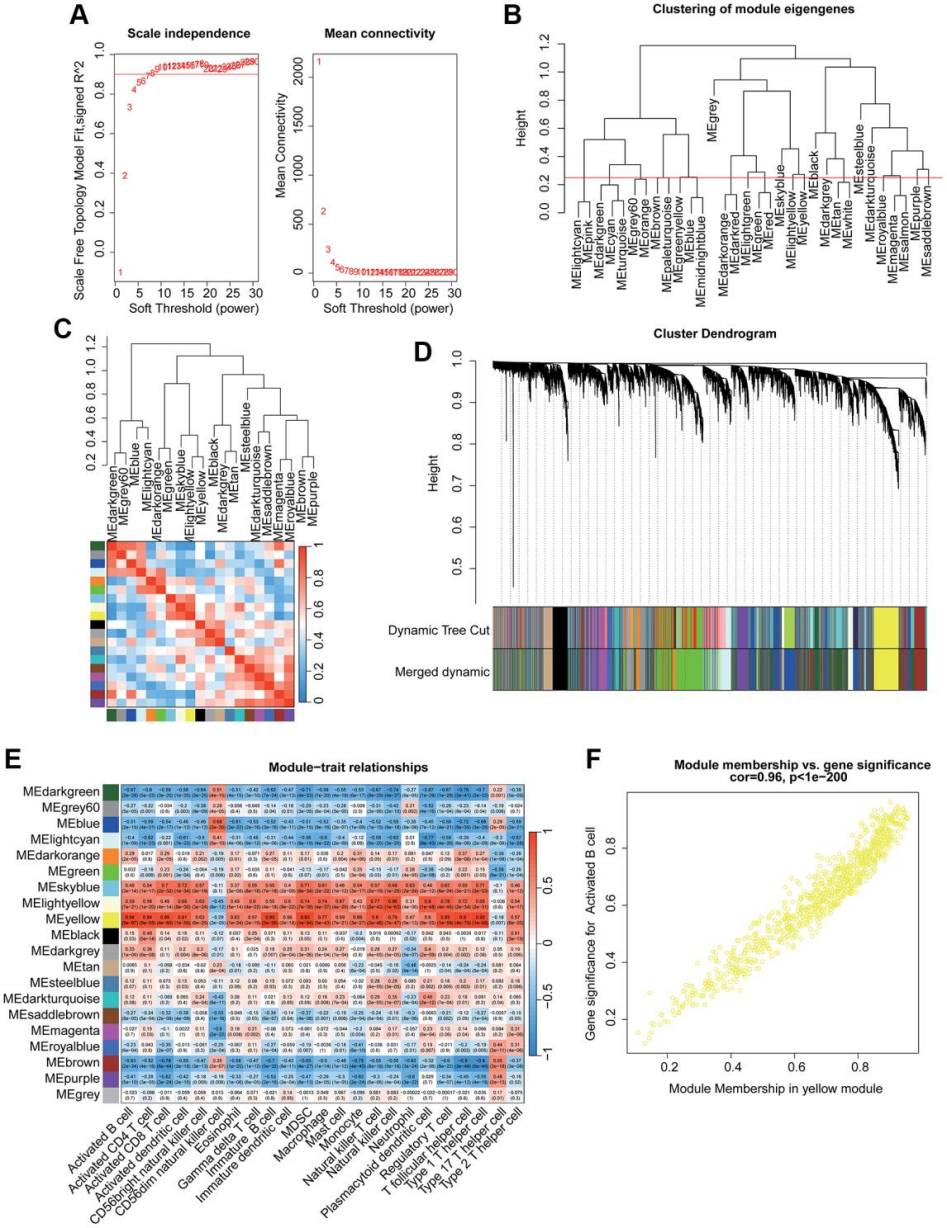
**Identification the immune cell subtype related gene module for UC via WGCNA.** (**A**) Identification of the optimal soft threshold (β) to develop the WGCNA. (**B**) Clustering of each gene module. (**C**) Correlation evaluation of each gene module. (**D**) Generation of the unique gene module via dynamic tree cut. (**E**) Correlation analysis of each gene module and 23 immune cell subtypes. (**F**) Relationship of module membership in yellow module and gene significance.

Two independent cohorts (GSE48634 and GSE92415) were included to identify the most pivotal immune cell related gene module associated with UC. After filtering the transcriptome data, all samples were clustered to exclude outlier samples. With the scale free topology model (R^2^) set at 0.85, the soft threshold (β = 5) was set to establish the WGCNA (Figure 1A). Under the height of clustering tree set at 0.25, we clustered each gene module and the correlation result illustrated a weak relationship of each gene module (Figure 1B, 1C). The gene modules were subsequently cut by dynamic tree and merged the gene modules for the final analysis (Figure 1D). After the correlation evaluation of each gene module and 23 immune cell subtypes, a strong correlation was observed in most gene modules and immune cell subtypes, especially the yellow gene module and activated B cell (Figure 1E). The scatter dot analysis revealed a strong correlation between the module membership and gene significance in yellow module (r = 0.96, *p* < 1e−200), and this gene module was identified as the pivotal activated B cell-related gene module for UC (Figure 1F).

### Identification of differential expression activated B cell-related gene signature and molecular pathway analysis

**Figure 2:**
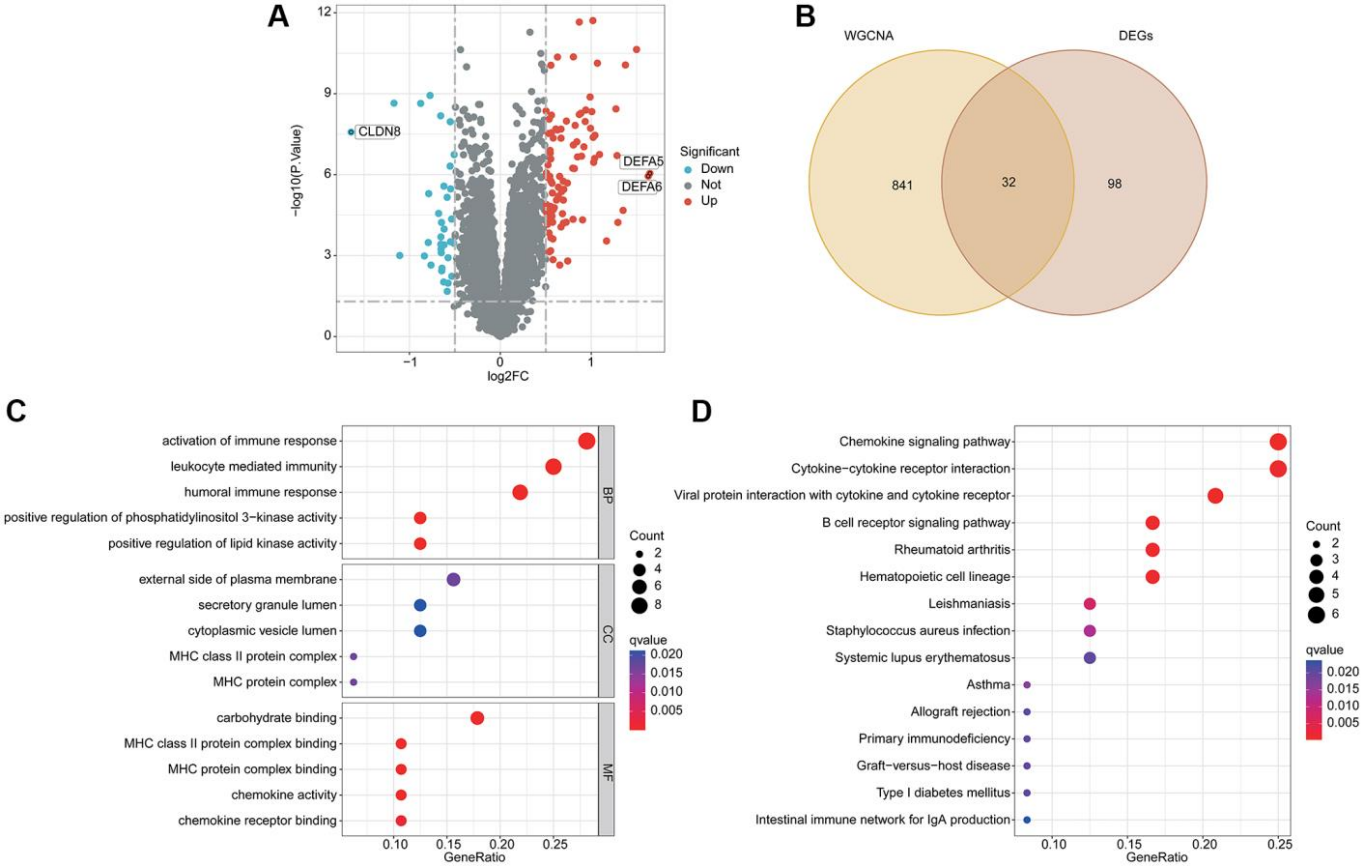
**Analysis of differential expression BRGs and potential molecular pathway exploration.** (**A**) Differential expression analysis by volcano plot. (**B**) Overlapping gene screening after WGCNA and differential expression analysis. (**C**) GO enrichment analysis. (**D**) The KEGG pathway analysis.

According to the differential expression analysis of HC and UC group, the DEGs were further identified via “limma” script (Figure 2A). Based on the WGCNA and differential expression analysis, a total of 32 pivotal activated B cell-related gene (BRGs) signatures were identified (Figure 2B). GO enrichment analysis suggested that the pivotal BRGs were involved in the activation of immune response, leukocyte mediated immunity, humoral immune response, external side of plasma membrane and carbohydrate binding (Figure 2C). The KEGG pathway result illustrated that the pivotal BRGs were related to chemokine signaling pathway, cytokine−cytokine receptor interaction, viral protein interaction with cytokine and cytokine receptor and B cell receptor signaling pathway (Figure 2D).

### Investigation of BRGs related diagnostic biomarker via machine learning algorithm

**Figure 3:**
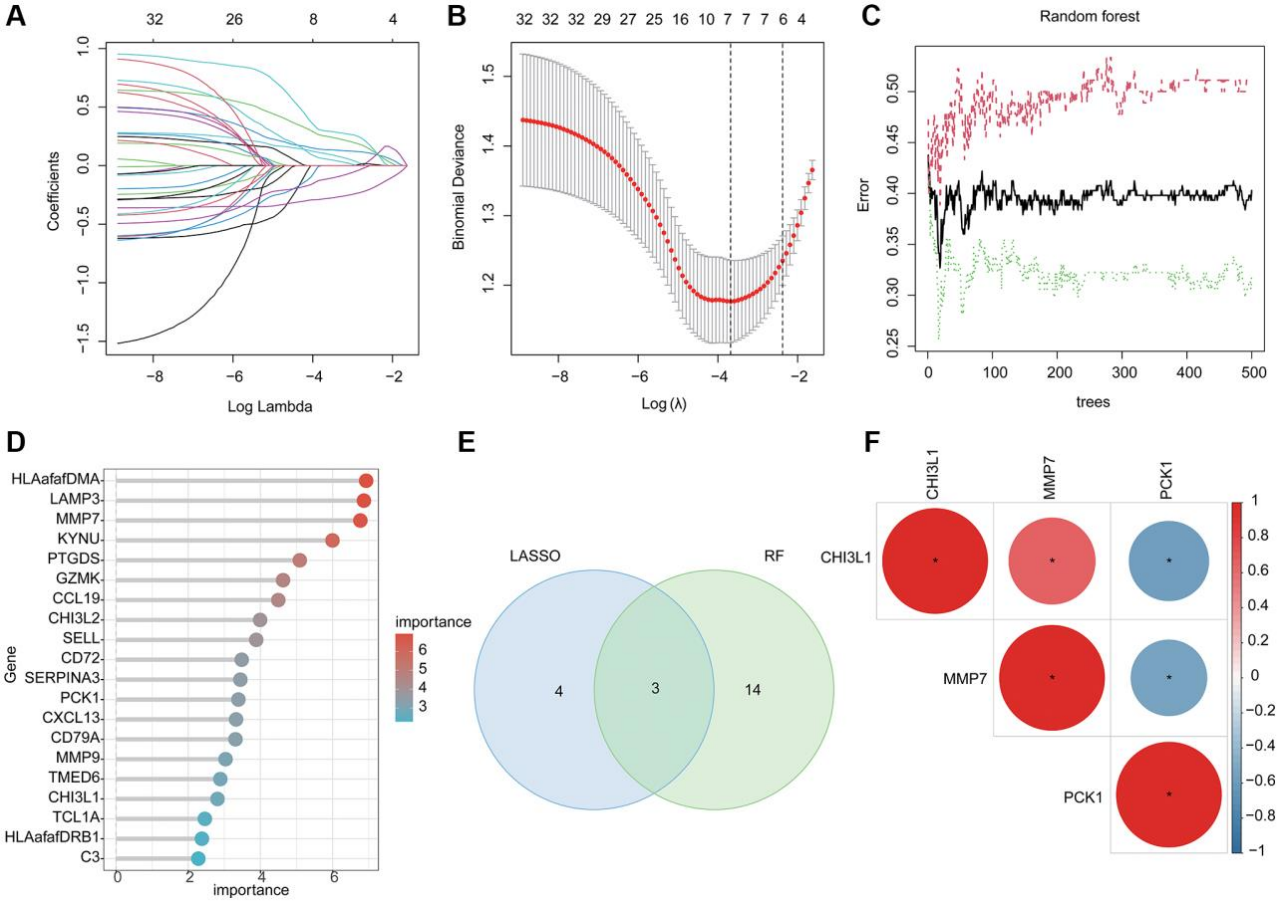
**Machine learning based BRGs diagnostic biomarkers identification.** (**A**, **B**) LASSO analysis of 32 pivotal BRGs related gene signatures. (**C**, **D**) The importance calculation of BRGs related gene signatures via RF algorithm. (**E**) Identification of BRGs related diagnostic biomarkers via RF and LASSO. (**F**) Potential correlation analysis of 3 BRGs related diagnostic biomarkers.

LASSO and RF algorithms were utilized to identify the BRGs related diagnostic biomarker for UC. On the basis of LASSO analysis, 7 BRGs related gene signatures were selected according to the minimum log lambda and optimal coefficient (Figure 3A, 3B). Utilizing the RF algorithm, the importance of 32 pivotal BRGs related gene signatures was calculated and 17 important variables were selected (Figure 3C, 3D). Finally, 3 overlapping BRGs related gene signatures were obtained via RF and LASSO algorithms and were considered as the BRGs related diagnostic biomarkers for UC (Figure 3E). The correlation analysis revealed that CHI3L1 was positively related to MMP7 and negatively related to PCK1; MMP7 was negatively correlated with PCK1 (Figure 3F).

### Diagnostic effectiveness evaluation of BRGs related biomarker and nomogram establishment

**Figure 4:**
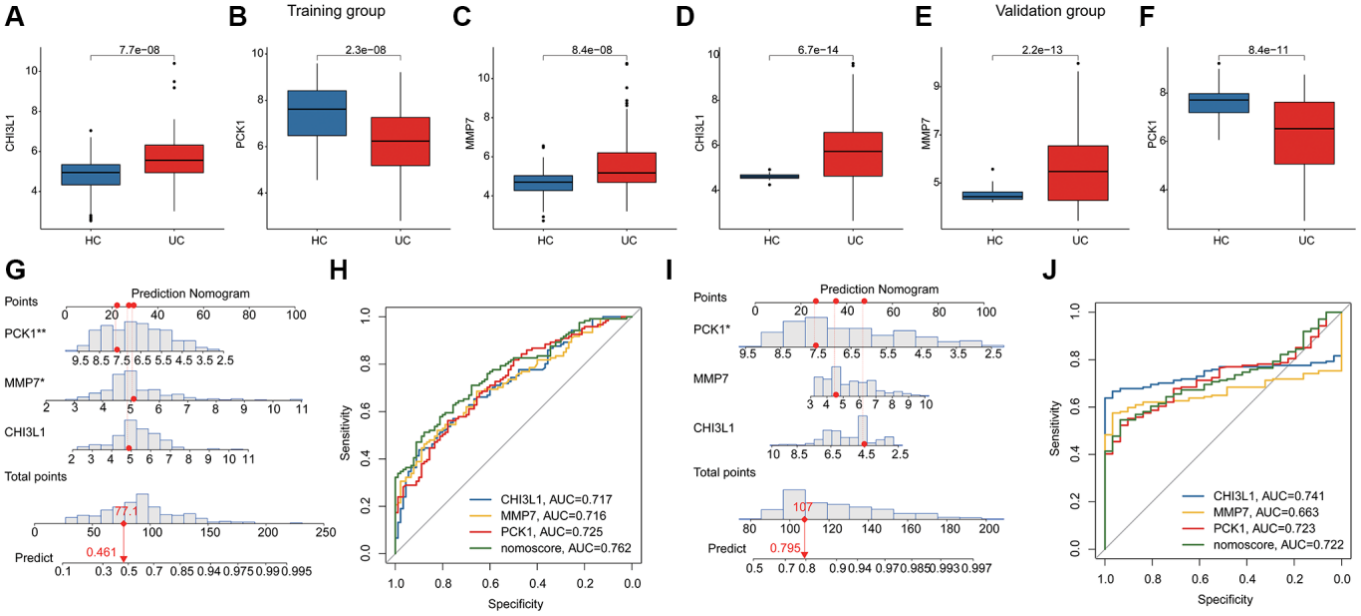
**Diagnostic ability estimation and nomogram model development of BRGs related biomarkers in UC.** (**A**–**F**) The expression level of BRGs related biomarkers of HC and UC samples in the training and validation subgroups. (**G**) Nomogram development of CHI3L1, PCK1, MMP7 in the training subgroup. (**H**) ROC analysis of BRGs related biomarkers and nomogram in the training subgroup. (**I**) Nomogram development of CHI3L1, PCK1, MMP7 in the validation subgroup. (**J**) ROC analysis of BRGs related biomarkers and nomogram in the validation subgroup.

Two independent cohort (GSE179285 and GSE107499) were utilized to validate the expression and diagnostic ability of the BRGs related biomarkers for UC. In the training subgroup, the expressions of CHI3L1 and MMP7 were observed to be overexpressed in the UC group, whereas the expression of PCK1 was down-regulated in the UC group. Notably, the expression level of the BRGs related biomarkers in the validation subgroup was consisted with the training subgroup (Figure 4A–4F). Based on the expression profile of 3 BRGs related biomarkers, we subsequently developed a nomogram model to evaluate the diagnostic ability of BRGs related biomarkers in both training and validation subgroups. As shown in Figure 4G–4J, the AUCs of CHI3L1, PCK1, MMP7 and nomogram were 0.717, 0.725, 0.716 and 0.762 in the training subgroup and 0.741, 0.723, 0.663 and 0.722 in the validation subgroup.

### Potential association of BRGs related biomarkers and immune infiltration characterization

**Figure 5:**
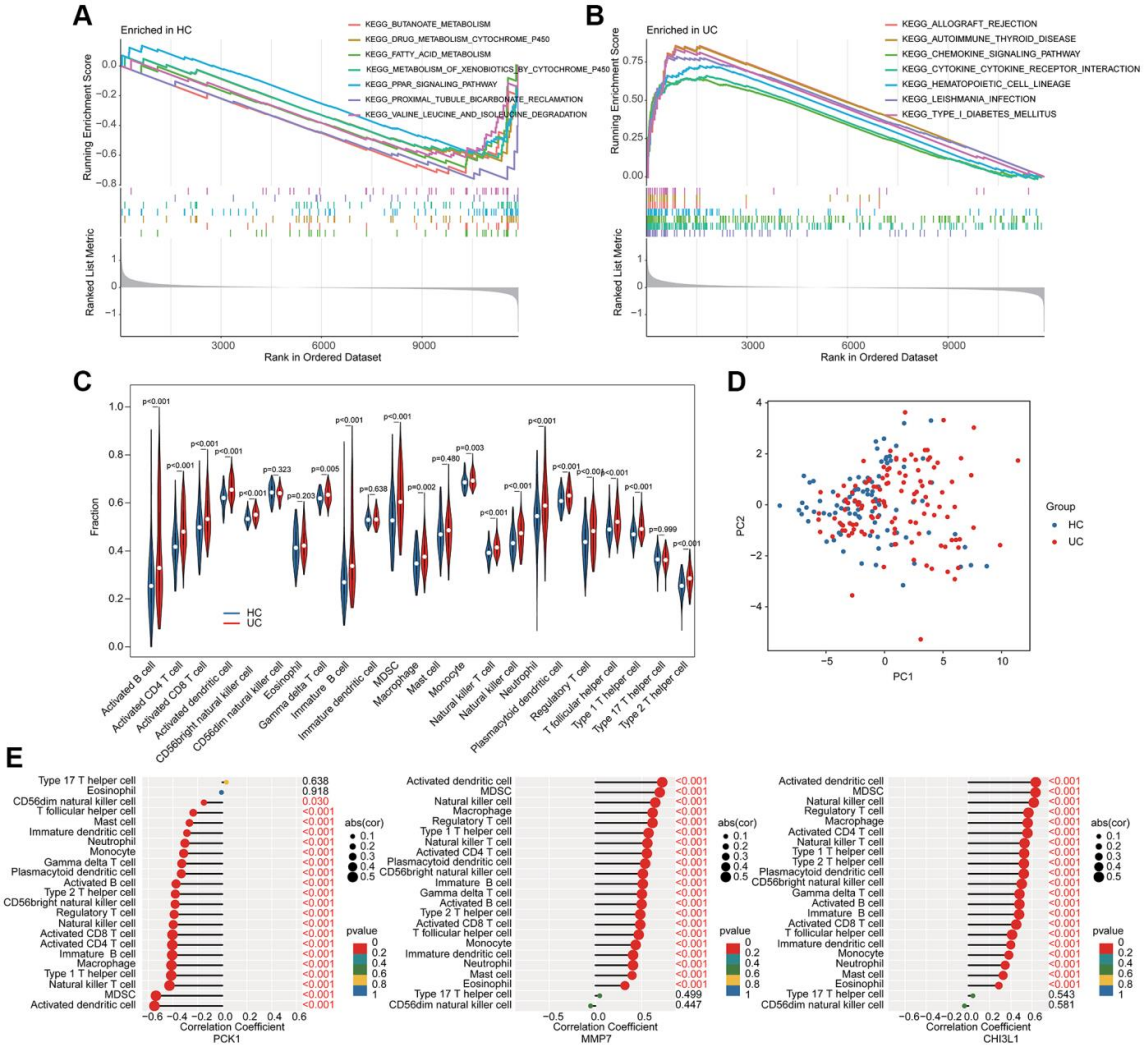
**GSEA analysis and association analysis of immune infiltration characterization and BRGs related biomarkers.** GSEA estimation of (**A**) HC group and (**B**) UC group. (**C**) Immune infiltration characterization evaluation. (**D**) PCA analysis of immune cells between HC and UC groups. (**E**) Potential association of 3 BRGs related biomarkers and immune infiltration characterization.

We further conducted a GSEA analysis to reveal the potential molecular pathways. In HC group, the metabolic pathways related to some small molecules were significantly upregulated, involving butanoate metabolism, drug metabolism cytochrome p450 and fatty acid metabolism (Figure 5A). Additionally, a series of immune related molecular pathways was observed significantly upregulated in the UC group, such as chemokine signaling pathway, cytokine-cytokine receptor interaction and hematopoietic cell lineage (Figure 5B). The quantitative data of immune infiltration characterization suggested that most of the immune cells had higher expression levels in the UC group, such as activity B cell, CD4^+^ T cell, CD8^+^ T cell, activated dendritic cell, CD56 bright natural killer cell, immature B cell, MDSC, macrophage, monocyte, and natural killer T cell (Figure 5C). Principal component analysis (PCA) of the immune infiltration characterization revealed a clear distinction between HC and UC groups (Figure 5D). Correlation analysis of BRGs related biomarkers and immune infiltration characterization illustrated that the PCK1 was negatively associated with most immune cells, whereas MMP7 and CHI3L1 were positively associated with most immune cells (Figure 5E). Collectively, these results demonstrated a significant difference in immune infiltration characterization between the HC and UC groups and the screened BRGs related biomarkers were closely associated with the immune infiltration.

### Identification of molecular subgroup and immune infiltration analysis for UC

**Figure 6:**
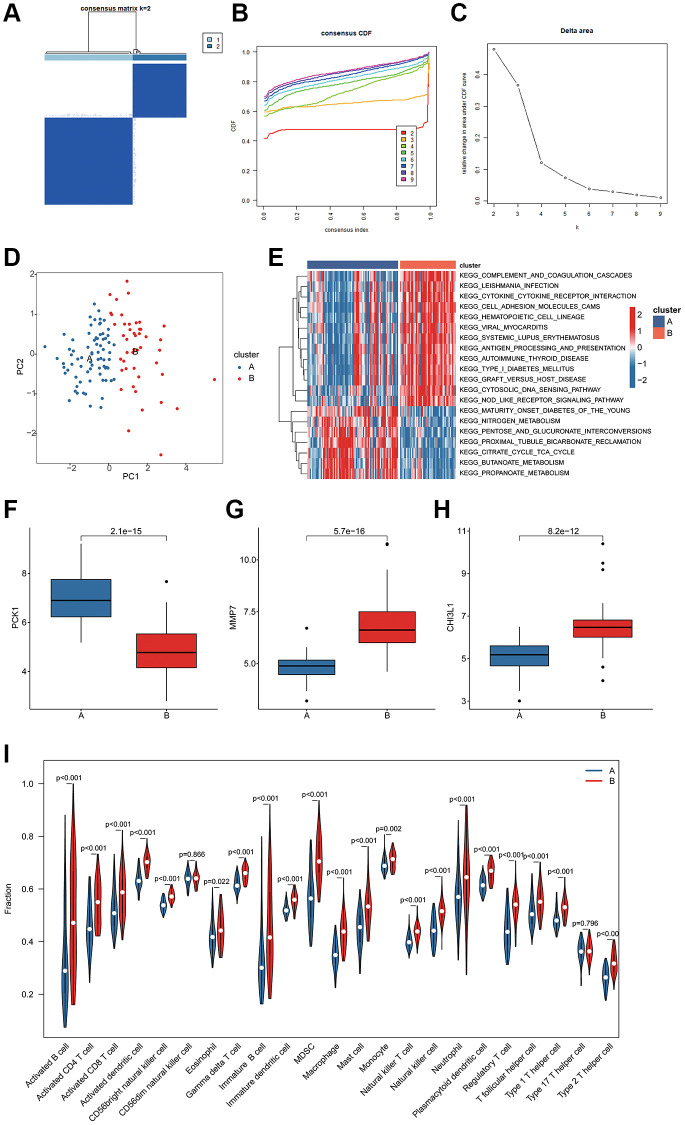
**BRGs-based molecular subgroups identification and immune infiltration evaluation.** (**A**–**C**) BRGs-based molecular subgroups generation. (**D**) PCA analysis of cluster A and B. (**E**) GSVA analysis of molecular pathways. (**F**–**H**) Expression profile of 3 BRGs related biomarkers of BRGs-based cluster subgroups. (**I**) Immune infiltration characterization of BRGs-based cluster subgroups.

Based on the BRGs related biomarkers, we conducted a consensus clustering analysis of UC samples. Under the optimal classification of K = 2, the UC samples were classified into 2 molecular subgroups (Figure 6A–6C). The PCA diagram displayed a distinct distribution pattern of BRG-based cluster A and cluster B based on the BRGs related biomarkers (Figure 6D). The GSVA result suggested that a series of immune related molecular pathways were greatly upregulated in the cluster B, while some metabolism related molecular pathways were upregulated in the cluster A (Figure 6E). The BRGs related biomarkers expression profile revealed that the expression of PCK1 was down-expressed of UC samples in the cluster B, whereas the expressions of MMP7 and CHI3L1 were over-expressed of UC samples in cluster B (Figure 6F–6H). Immune infiltration analysis of BRGs-based molecular subgroups illustrated that the UC samples in the cluster B had higher immune infiltration level, including activity B cell, CD4^+^ T cell and CD8^+^ T cell (Figure 6I).

### *In vitro* qRT-PCR validation of BRGs related biomarkers

**Figure 7:**
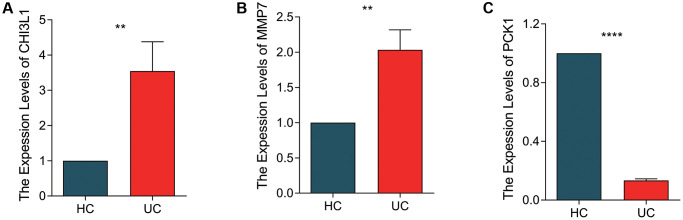
**qRT-PCR validation of BRGs related biomarkers in NCM460 cell line and LPS-induced NCM460 cell line.** The quantitative statistical analysis of expression levels of (**A**) CHI3L1, (**B**) MMP7 and (**C**) PCK1 in NCM460 and LPS-induced NVM460 cell lines. ^*^p < 0.05, ^**^p < 0.01, ^***^p < 0.001.

We further validated the expressions of 3 screened BRGs *in vitro* by LPS-induced NCM460 cell line model. As shown in Figure 7, the mRNA expression of CHI3L1 and MMP7 were significantly overexpressed, while PCK1 was obviously lower in the UC group.

## DISCUSSION

As a clinical disease with poor therapeutic effect, the further study of UC has positive clinical significance. In this study, we screened B cell-associated UC diagnostic markers. In addition, immunosignature analysis and pathway enrichment analysis of UC patients provide possible clues for the involvement of UC prognosis.

In this study, we screened out three diagnostic markers of UC. Similar to the results of another multicenter study of intestinal gene expression differences, we also identified MMP7 as a differential upregulator of UC [21]. As a member of matrix metalloproteinase (MMP), MMP7 has proteolytic activity against a variety of substrates [22]. Defects in specific components of the mucosal barrier are one of the specific structural changes in UC patients and allow chronic mucosal inflammation to persist [23]. In UC, it has been reported that MMP-7 degraded the tight junction protein Claudin-7 in epithelial cells, which damaged the intestinal epithelial barrier and increases inflammation. Treatment with MMP-7 monoclonal antibody improved intestinal barrier function and reduced inflammation in rats [24]. This suggests MMP-7 as a potential therapeutic target for IBD.

Chitinase 3-like 1 (CHI3L1) belongs to the glycohydrolase 18 family of chitinases. CHI3L1 has been reported to enhance the ability of bacteria to adhere to and invade colon epithelial cells to exacerbate intestinal inflammation [25, 26]. Therefore, its expression is elevated in IBD patients and is accompanied by increasing disease activity [25, 27]. Some studies have even suggested that loss of tolerance to CHI3L1 is a characteristic of UC [28]. In addition, similar to MMP7, CHI3L1 expression is increased during the progression of colitis-associated carcinoma (CAC) [29]. Oxidative damage caused by CHI3L1 by inhibiting the increase in reactive oxygen species (ROS) induced by caffeine may be one of the reasons [30]. CHI3L1 was reported to be associated with infliximab/adalimumab and vedolizumab treatment response [31]. All these suggest the potential of CHI3L1 as an ideal target for clinical intervention.

We also identified phosphoenolpyruvate carboxykinase 1 (PCK1) as a differentially regulated gene for UC. PCK1, an enzyme involved in glucose production, also regulates adipogenesis and is associated with hepatic steatosis [32]. Although it has not been reported in depth in UC, there are clues to its role at UC. PCK1 contributes to M1 polarization in macrophages, suggesting the role of PCK1 in inhibiting inflammation [33]. In mouse models, the latest reports show that PCK1 knocked-out mice increased inflammatory infiltration and caused high levels of TNF-α. At the same time, PCK1 deficiency through the PI3K/AKT/PDGF axis significantly increased mRNA levels of genes associated with inflammation, which is in line with our result [34]. Given that the regulation of the PI3K/AKT axis has become a hot topic in IBD treatment research [35], PCK1 is expected to receive further attention.

Our results showed that molecular subtyping of BRGs showed that in addition to immune-related biological processes, including antigen processing and presentation, graft versus host disease, a variety of metabolism-related pathways were also enriched to be related to risk stratification, such as nitrogen metabolism, TCA, butanoate metabolism, propanoate metabolism. To satisfy cell growth and proliferation, the activation process of immune cells is accompanied by metabolic reprogramming [36]. In addition, metabolic reprogramming has also been shown to affect B cell differentiation [37]. Their interaction creates a complex link between BRGs and metabolism. Although there is some evidence suggesting the role of metabolic processes such as TCA in UC [38], the study of these metabolic processes in UC is still unveiled. Additionally, we have not been able to find evidence that B cells are involved in these metabolic pathways. But at the very least, our study sheds light on the role of metabolic pathways in UC risk stratification and the potential involvement of B cells in this process.

We observed a higher concentration of myeloid suppressor cells (MDSCs) in patients with UC. It was reported that increased frequency of MDSC was observed in peripheral blood of patients with IBD and could directly regulate IBD progression by regulating T cell function [39]. In addition, highly expressed MDSC is associated with worsening of IBD and an increased likelihood of cancer progression [40]. The mechanism may be that MDSC produces IL-10, which in turn promotes cancer initiation by regulating the STAT3-DNMT3b-IRF8 axis [41]. This may explain the increased incidence of colon cancer in UC patients. The intervention of MDSC to control UC disease progression has clinical application potential.

Another immune cell whose expression was significantly different in the immune microenvironment and significantly correlated with the expression level of the screened UC diagnostic markers in this study was dendritic cells (DCs). DC regulates responses to the gut microbiota by acting as a bridge between innate and adaptive immune responses, and is one of the core players in UC by influencing the mucosal immune system [42]. The level of DC expression is significantly decreased in patients with acute UC and correlated with disease activity [43]. TNF-a, IL-6, and IL-8 secreted by plasmacytoid dendritic cells (pDC) are also significantly increased in UC patients [44]. In addition, the CD103^+^ DC subtype has an impaired ability to generate Treg cells while inducing a Th1/Th2/Th17 immune response that promotes UC development [45]. Therefore, a number of DC-associated proteins associated with UC genetic susceptibility have been identified as potential therapeutic targets [46]. At the same time, various immune-related treatments such as autophagy and probiotic regulation have also been shown to be associated with DC participation [47]. Our results showed that the three selected targets were significantly correlated with the expression level of DC, which also suggested the necessity of further exploring the function of DC in UC.

In summary, we found a strong correlation between activated B cells and UC immune cell-related gene modules, and obtained three BRGs as UC-related diagnostic biomarkers. Since the biomarker screening process in this study was based on public database, there was more of a correlation analysis than a causation analysis. Although *in vitro* experiments preliminarily verified the correlation between markers and UC to some extent, limited to conditions, the exact relationship between the screened markers and the established UC molecular subtypes cannot be established, and their molecular mechanisms were not further explored. Our results at least provide new evidence and potential intervention targets for the role of B cells in UC. In the future, further clinical validation and *in vitro* and *in vivo* experimental analysis will help to deepen this study.

## Supplementary Materials

Supplementary Table 1
