# Correction for: Machine learning-based B cell-related diagnostic biomarker signature and molecular subtypes characteristic of ulcerative colitis

**DOI:** 10.18632/aging.206209

**Published:** 2025-02-28

**Authors:** Guo-Liang Wu, Li Li, Xiao-Yao Chen, Wei-Feng Zhang, Jun-Bo Wu, Xiaoning Yu, Hong-Jin Chen

**Affiliations:** 1 First Clinical Medical College, Nanjing University of Chinese Medicine, Nanjing, Jiangsu 210023, China; 2 Department of Anorectal Section, The First Affiliated Hospital of Shandong First Medical University, Jinan, Shandong 250014, China; 3 Department of Endocrinology, The Affiliated Hospital of Shandong University of Traditional Chinese Medicine, Jinan, Shandong 250014, China; 4 Department of Colorectal Surgery, The Affiliated Hospital of Nanjing University of Chinese Medicine, Nanjing, Jiangsu 210029, China; 5 Department of Anorectal Section, The Affiliated Hospital of Xuzhou Medical University, Xuzhou, Jiangsu 221000, China; 6 Department of Colorectal Surgery, Hengyang Central Hospital, Hengyang, Hunan 421001, China; 7 Department of Geriatrics, Hematology and Oncology Unit, Qilu Hospital of Shandong University, Jinan, China

**Keywords:** ulcerative colitis, B cell-related genes, diagnostic biomarker, immune infiltration, machine learning

**This article has been corrected:** The authors found that there was an error in the published affiliation for the corresponding author, Hong-Jin Chen. The correct affiliation is “^4^Department of Colorectal Surgery, The Affiliated Hospital of Nanjing University of Chinese Medicine, Nanjing, Jiangsu 210029, China.”

The full list of authors with their correctly assigned affiliations is provided below:

**Guo-Liang Wu^1,2^, Li Li^3^, Xiao-Yao Chen^2^, Wei-Feng Zhang^4,5^, Jun-Bo Wu^6^, Xiaoning Yu^7^, Hong-Jin Chen^4^**

^1^First Clinical Medical College, Nanjing University of Chinese Medicine, Nanjing, Jiangsu 210023, China

^2^Department of Anorectal Section, The First Affiliated Hospital of Shandong First Medical University, Jinan, Shandong 250014, China

^3^Department of Endocrinology, The Affiliated Hospital of Shandong University of Traditional Chinese Medicine, Jinan, Shandong 250014, China

^4^Department of Colorectal Surgery, The Affiliated Hospital of Nanjing University of Chinese Medicine, Nanjing, Jiangsu 210029, China

^5^Department of Anorectal Section, The Affiliated Hospital of Xuzhou Medical University, Xuzhou, Jiangsu 221000, China

^6^Department of Colorectal Surgery, Hengyang Central Hospital, Hengyang, Hunan 421001, China

^7^Department of Geriatrics, Hematology and Oncology Unit, Qilu Hospital of Shandong University, Jinan, China

