# Supplementary material for: Machine learning-based B cell-related diagnostic biomarker signature and molecular subtypes characteristic of ulcerative colitis
Source: Aging (Albany NY). 2024 Feb 5;16(3):2774–88. doi: 10.18632/aging.205510 (PMC10911385; doi:10.18632/aging.205510)
Supplement: Supplementary Table 1 [file aging-16-205510-s001.pdf]

SUPPLEMENTARY TABLE

Supplementary Table 1. The gene marker of the 23 types of immune cell.

Activated B cell: ADAM28 CD180 CD79B BLK CD19 MS4A1 TNFRSF17 IGHM GNG7 MICAL3 SPIB HLA-DOB IGKC PNOC FCRL2 BACH2 CR2 TCL1A AKNA ARHGAP25 CCL21 CD27 CD38 CLEC17A CLEC9A CLECL1

Activated CD4 T cell: AIM2 BIRC3 BRIP1 CCL20 CCL4 CCL5 CCNB1 CCR7 DUSP2 ESCO2 ETS1 EXO1 EXOC6 IARS ITK KIF11 KNTC1 NUF2 PRC1 PSAT1 RGS1 RTKN2 SAMSN1 SELL TRAT1

Activated CD8 T cell: ADRM1 AHSA1 C1GALT1C1 CCT6B CD37 CD3D CD3E CD3G CD69 CD8A CETN3 CSE1L GEMIN6 GNLY GPT2 GZMA GZMH GZMK IL2RB LCK MPZL1 NKG7 PIK3IP1 PTRH2 TIMM13 ZAP70

Activated dendritic cell: ABCD1 C1QC CAPG CCL3L3 CD207 CD302 ATP5B ATP5L ATP6V1A BCL2L1 C1QB SNURF SPCS3 CCNA1 CEACAM8 NOS2 SRA1 TNFRSF6B TREM1 TREML1 RHOA SLC25A37 TNFSF14 TREML4 VNN2 XPO6 CLEC4C TNFAIP2 UBD ACTR3 RAB1A SLA HLA-DQA2 SIGLEC5 SLAMF9

CD56bright natural killer cell: ABAT C11orf75 C5orf15 CDHR1 DCAF12 DYNLL1 GPR137B HCP5 HDGFRP2 KRT86 MLST8 ELMOD3 ENTPD5 FAM119A FAM179A CLIC2 COX7A2L CREB3L4 CSF1 CSNK2A2 CSTA CSTB CTPS CTSD FST GATA2 GMPR HDC HEY1 HOXA1 HS2ST1 HS3ST1 BCL11B CDH3 MYL6B NAA16 ClQA ClQB CYP27B1 EIF3M

CD56dim natural killer cell: CYP27A1 DDX55 DYRK2 RPL37A NOTCH3 AKR7A3 GPRC5C GRIN1 HLA-E PORCN PSMC4 UPP1 IL21R KIR2DS1 KIR2DS2 KIR2DS5

Eosinophil: GIPR KRT18P50 LRMP FOSB RRP12 GPR183 NR4A3 ST3GAL6 DEPDC5 PDE6C PKD2L2 GPR65 IL5RA P2RY14 DACH1 DAPK2 EMR3

Gamma delta T cell: ACP5 AQP9 BTN3A2 C1orf54 CARD8 CCL18 CD209 CD33 CD36 CDK5 IL10RB KLRF1 LGALS1 MAPK7 KLHL7 KRT80 LAMC1 LCORL LMNB1 MEIS3P1 MPL FABP1 FABP5 FADD MFAP3L MINPP1 RPS24 RPS7 RPS9 DBNL CCL13

Immature B cell: CD22 CYBB FAM129C FCRL1 FCRL3 FCRL5 FCRLA HDAC9 HLA-DQA1 HVCN1 KIAA0226 NCF1 NCF1B P2RY10 SP100 TXNIP STAP1 TAGAP ZCCHC2

Immature dendritic cell: ACADM AHCYL1 ALDH1A2 ALDH3A2 ALDH9A1 ALOX15 AMT ARL1 ATIC ATP5A1 CAPZA1 LILRA5 RDX RRAGD TACSTD2 INPP5F RAB38 PLAU CSF3R SLC18A2 AMPD2 CLTB C1orf162

MDSC: CCR2 CD14 CD2 CD86 CXCR4 FCGR2A FCGR2B FCGR3A FERMT3 GPSM3 IL18BP IL4R ITGAL ITGAM PARVG PSAP PTGER2 PTGES2 S100A8 S100A9

Macrophage: AIF1 CCL1 CCL14 CCL23 CCL26 CD300LB CNR1 CNR2 EIF1 EIF4A1 FPR1 FPR2 FRAT2 GPR27 GPR77 RNASE2 MS4A2 BASP1 IGSF6 HK3 VNN1 FES NPL FZD2 FAM198B HNMT SLC15A3 CD4 TXNDC3 FRMD4A CRYBB1 HRH1 WNT5B

Mast cell: ADAMTS3 CPA3 CMA1 CTSG ARHGAP15 CPM FCN1 FTL HSPA6

ITGA9 RNASE3 S100A4 SIGLEC8 SLC6A4 PTGS2 EGR3 PILRA

Monocyte: ASGR2 CFP ASGR1 CD1D UPK3A ACTG1 ANXA5 ATP6V1B2 CFL1 DAZAP2 CTBS EMR4P HIVEP2 MARCKSL1 MBP MMP15 PNPLA6 TMBIM6 PQBP1 TEX264 IKZF1

Natural killer T cell: BTN2A2 CD101 CD109 CNPY3 CNPY4 CREB1 CRTC2 CRTC3 CSF2 KLRC1 FUT4 ICAM2 IL32 LAMP2 LILRB5 KLRG1 HSPA4 HSPB6 ISM2 ITIH2 KDM4C KIR2DS4 KIRREL3 SDCBP NFATC2IP MICB KIR2DL1 KIR2DL3 KIR3DL1 KIR3DL2 NCR1 FOSL1 TSLP SLC7A7 SPP1 TREM2 UBASH3A YBX2 CCDC88A CLEC1A THBD PDPN VCAM1 EMR1

Natural killer cell: AKT3 AXL BST2 CDH2 CRTAM CSF2RA CTSZ CXCL1 CYTH1 DAXX DGKH DLL4 DPYD ERBB3 F11R FAM27A FAM49A FASLG FCGR1A FN1 FSTL1 FUCA1 GBP3 GLS2 GRB2 LST1 BCL2 CDC5L FGF18 FUT5 FZR1 GAGE2 IGFBP5 KANK2 LDB3

Neutrophil: CREB5 CDA CHST15 S100A12 APOBEC3A CASP5 MMP25 HAL C1orf183 FFAR2 MAK CXCR1 STEAP4 MGAM BTNL8 CXCR2 TNFRSF10C VNN3

Plasmacytoid dendritic cell: CBX6 DAB2 DDX17 HIGD1A IDH3A IL3RA MAGED1 NUCB2 OFD1 OGT PDIA4 SERTAD2 SIRPA TMED2 ENG FCAR IGF1 ITGA2B GABARAP GPX1 KRT23 PROK2 RALB RETNLB RNF141 SEC14L1 SEPX1 EMP3 CD300LF ABTB1 KLHL21 PHRF1

Regulatory T cell: CCL3L1 CD72 CLEC5A FOXP3 ITGA4 L1CAM LIPA LRP1 LRRC42 MARCO MMP12 MNDA MRC1 MS4A6A PELO PLEK PRSS23 PTGIR ST8SIA4 STAB1

T follicular helper cell: B3GAT1 CDK5R1 PDCD1 BCL6 CD200 CD83 CD84 FGF2 GPR18 CEBPA CECR1 CLEC10A CLEC4A CSF1R CTSS DMN DPP4 LRRC32 MC5R MICA NCAM1 NCR2 NRP1 PDCD1LG2 PDCD6 PRDX1 RAE1 RAET1E SIGLEC7 SIGLEC9 TYRO3 CHST12 CLIC3 IVNS1ABP KIR2DL2 LGMN

Type 1 T helper cell: CD70 TBX21 ADAM8 AHCYL2 ALCAM B3GALNT1 BBS12 BST1 CD151 CD47 CD48 CD52 CD53 CD59 CD6 CD68 CD7 CD96 CFHR3 CHRM3 CLEC7A COL23A1 COL4A4 COL5A3 DAB1 DLEU7 DOC2B EMP1 F12 FURIN GAB3 GATM GFPT2 GPR25 GREM2 HAVCR1 HSD11B1 HUNK IGF2 RCSD1 RYR1 SAV1 SELE SELP SH3KBP1 SIT1 SLC35B3 SIGLEC10 SKAP1 THUMPD2 TIGIT ZEB2 ENC1 FAM134B FBXO30 FCGR2C STAC LTC4S MAN1B1 MDH1 MMD RGS16 IL12A P2RX5 CD97 ITGB4 ICAM3 METRNL TNFRSF1A IRF1 HTR2B CALD1 MOCOS TRAF3IP2 TLR8 TRAF1 DUSP14

Type 17 T helper cell: IL17A IL17RA C2CD4A C2CD4B CA2 CCDC65 CEACAM3 IL17C IL17F IL17RC IL17RE IL23A ILDR1 LONRF3 SH2D6 TNIP2 ABCA1 ABCB1 ADAMTS12 ANK1 ANKRD22 B3GALT2 CAMTA1 CCR9 CD40 GPR44 IFT80

Type 2 T helper cell: ASB2 CSRP2 DAPK1 DLC1 DNAJC12 DUSP6 GNAI1 LAMP3 NRP2 OSBPL1A PDE4B PHLDA1 PLA2G4A RAB27B RBMS3 RNF125 TMPRSS3 GATA3 BIRC5 CDC25C CDC7 CENPF CXCR6 DHFR EVI5 GSTA4 HELLS IL26 LAIR2
